# Supplementary material for: Longitudinal monitoring of twenty homes reveals spatiotemporal dynamics which require new models of discomfort and thermostat use
Source: Sci Rep. 2026 Jan 10;16:2888. doi: 10.1038/s41598-025-32727-y (PMC12827413; doi:10.1038/s41598-025-32727-y)
Supplement: Supplementary file 1 — Supplementary Information. [file 41598_2025_32727_MOESM1_ESM.pdf]

Longitudinal Monitoring of Twenty Homes Reveals Spatiotemporal Dynamics which Require New Models of Discomfort and Thermostat use

SungKu Kang[1,2+], Maharshi Pathak[2+], Kunind Sharma[2+], Emily Casavant[2], Katherine Bassett[2], Misha Pavel[3], David Fannon[2,4], Michael Kane[2,*]

[1] Korea Aerospace University, Department of Mechanical and Aircraft System Engineering, Goyang-si, Gyeonggi-do, 10540, Republic of Korea
[2] Northeastern University, Civil and Environmental Engineering Department, Boston, MA 02115 United States
[3] Northeastern University, Khoury College of Computer Sciences, Boston, MA 02215, United States
[4] Northeastern University, School of Architecture, Boston, MA 02215, United States
* mi.kane@northeastern.edu
+ these authors contributed equally to this work and are listed alphabetically

Appendix

Summary of Home Characteristics

Home No.	State	City	A_{floor} (ft^2)	Home Age (yrs)	Renovation/ Additions	No. of Floors	Years of Occupancy	No. of Occupants	Pre-study Thermostat Type	No. of Thermostats	Equivalent Leakage Area – ELA (ft^2)
1	MA	Canton	2,054	72	2004	2	25	3	Programmable	One (1)	7.8
2	MA	Worcester	2,050	36	N/A	2	13	5	Smart	One (1)	3.5
3	MA	Worcester	2,051	21	2016	3	5	5	Smart	Three (3)	3.8
4	MA	Worcester	1,612	5	N/A	2	5	4	Smart	One (1)	1.56
5	MA	Worcester	2,285	30-40	N/A	2	5	5	Programmable	One (1)	3.1
6	MA	Medford	1,746	20	N/A	3	5	4	Smart	Two (2)	2.8
7	MA	Medford	2,042	122	2019	2	<1	2	Programmable	Two (2)	5.1
8	MA	Somerville	2,882	26	N/A	3	4	5	Programmable	One (1)	3.8
9	MA	Melrose	2,592	120+	N/A	4	20	6	Manual	One (1)	----
10	MA	Boxford	6,164	40	N/A	3	<1	5	Programmable	Two (2)	9.9
11	CO	Denver	1,618	17	N/A	2	2	4	Programmable	One (1)	1.5
12	CO	Lafayette	2,236	22	N/A	3	7	4	Smart	One (1)	0.68
13	CO	Lafayette	1,371	39	N/A	2	2	2	Programmable	One (1)	1.21
14	CO	Erie	2,253	17	N/A	1	12	2	Programmable	One (1)	1.53
15	CO	Lafayette		29	N/A	3	15	2	Smart	One (1)	2.14
16	CO	Lafayette		36	N/A	3	<1	4	Smart	One (1)	1.25
17	CO	Littleton	4,072	25	N/A	3	<1	3	Smart	One (1)	2.67
18	CO	Broomfield	2,670	13	N/A	2	<1	4	Programmable	Two (2)	0.78
19	CO	Erie	3,619	<1	N/A	3	<1	5	Smart	Two (2)	1.76
20	CO	Lafayette	3,389	26	N/A	2	12	5	Programmable	One (1)	1.64

Supplementary Table 1: Summary of participating home characteristics in Whole Energy Home (WEH) research project

#	T*max			T*max > 2				T*avg			T*avg > 2			
	count	mean	std	count	%	mean	std	count	mean	std	count	%	mean	std
1	10.7	3.0	1.8	7.0	65.4	3.7	1.9	10.7	1.7	0.8	3.0	27.6	2.7	0.7
2	6.7	5.3	2.3	6.6	98.9	5.3	2.3	6.7	4.0	1.4	6.5	97.0	4.0	1.4
3	10.8	3.4	3.1	6.3	57.8	4.9	3.3	10.8	2.2	2.2	3.8	35.3	4.4	2.5
4	2.7	5.0	3.6	2.2	84.2	5.7	3.5	2.7	3.5	2.3	1.9	72.5	4.2	2.3
5	11.0	4.0	2.6	8.0	72.1	5.1	2.3	11.0	1.9	1.3	4.5	40.9	3.1	1.2
6	6.8	4.6	2.8	6.1	90.6	4.9	2.7	6.8	2.7	1.6	3.9	57.0	3.6	1.6
7	12.5	2.9	1.8	8.4	66.9	3.6	1.7	12.5	1.9	1.2	4.1	32.5	3.1	1.3
8	10.4	3.0	1.7	8.2	78.9	3.5	1.7	10.4	2.0	0.9	4.6	44.7	2.7	0.9
9	10.8	5.4	3.8	8.7	81.0	6.4	3.6	10.8	2.5	1.8	5.8	53.8	3.7	1.6
10	10.6	4.6	3.1	9.1	86.3	5.1	3.1	10.6	3.1	2.9	5.2	49.4	4.9	3.2
11	7.3	5.1	3.6	6.7	93.0	5.4	3.6	7.3	3.1	1.7	5.8	79.9	3.5	1.7
12	8.9	2.6	1.2	5.7	63.7	3.3	0.8	8.9	1.6	0.9	2.6	28.9	2.8	0.6
13	10.6	5.2	3.3	8.8	83.3	6.0	3.1	10.6	3.7	2.3	7.9	74.7	4.5	2.1
14	9.7	3.3	1.7	7.8	80.6	3.8	1.5	9.7	2.4	1.2	6.1	62.8	3.1	0.9
15	8.4	4.0	2.6	6.4	76.8	4.8	2.5	8.4	2.5	1.6	4.6	54.4	3.5	1.5
16	10.4	4.3	2.9	8.1	78.1	5.2	2.7	10.4	2.6	1.7	6.0	57.5	3.6	1.4
17	10.6	3.5	1.9	8.3	78.4	4.2	1.5	10.6	2.2	1.1	6.4	60.1	2.8	0.7
18	9.1	3.3	1.4	8.4	92.6	3.5	1.3	9.1	2.3	0.8	5.9	65.3	2.7	0.6
19	9.5	5.0	2.9	8.5	89.4	5.4	2.8	9.5	2.4	1.3	5.5	58.3	3.2	1.1
20	9.5	6.0	2.2	9.3	98.0	6.1	2.1	9.5	4.2	1.8	8.5	90.2	4.5	1.7

Supplementary Table 2. Detailed statistics of spatial temperature variations across the home (**Figure 5**). Count is the thousands count of time series temperature samples. Mean (°*F)*, standard deviation (°*F)*, and percentage of time (%) that spatial temperature variations exceed 2°*F*. These statistics are calculated for T*max, the absolute difference at each sample time between the thermostat temperature reading and the remote sensor in an occupied room with the greatest temperature difference from the thermostat, and T*avg, the average of absolute difference between the thermostat temperature reading and each occupied room temperature at each sample time.

**Percentile**	**A** (min. / °F)	**B** (min.)	**RMSE**
50	1.269	54.22	7.18

Supplementary Table 3. Coefficients and Root Mean Square Error (RMSE) of the Linear Regression Curves Representing the 50th Percentile of MSCs. The table below provides the coefficients and RMSE values for the linear regression curves corresponding to the 50th percentile of manual setpoint changes (MSCs). These curves represent the relationship between the degree of manual setpoint change (DoMSC) and the time to manual setpoint change (TtMSC) within 150 minutes, as shown in Figure 11. The linear fitting function is expressed as $y = Ax + B$, where A represents the slope, and B represents the intercept.
